# Coronary Subclavian Steal Syndrome Unamenable to Angioplasty Successfully Managed with Subclavian-Subclavian Bypass

**DOI:** 10.1155/2012/784231

**Published:** 2012-04-09

**Authors:** Saad Tariq, Swosty Tuladhar, Edward Wingfield, Honesto Poblete

**Affiliations:** ^1^St Francis Medical Center Program, Seton Hall University, 210 Nottinghill lane, Hamilton, NJ 08619, USA; ^2^Hamilton Cardiology Associates, Hamilton, NJ 08619, USA; ^3^Robert Wood Johnson Hospital, Hamilton, NJ 08619, USA

## Abstract

*Purpose*. Coronary-subclavian steal syndrome (CSSS) is defined as a reversal of flow in a previously constructed internal mammary artery (IMA) coronary conduit, producing myocardial ischemia. We present a case of CSSS which could not be ameliorated with endovascular therapy and necessitated a subclavian-subclavian bypass. *Case Report*. 80-year-old Caucasian male with history of CABG presented with syncope. He had absent left-sided radial pulse with blood pressure being 60/40 on left arm and 130/80 on the right. He underwent cardiac catheterization for NSTEMI which showed patent left internal mammary artery graft to left anterior descending coronary artery with retrograde flow, and diagnosis of coronary subclavian steal syndrome was made. Complete occlusion of proximal left subclavian artery was identified. Percutaneous angioplasty failed because of calcified plaque causing 100% occlusion. Carotid doppler showed bilateral carotid artery disease. He finally underwent subclavian-subclavian bypass which resolved his condition. *Conclusion*. Subclavian-subclavian bypass is a successful alternative to carotid-subclavian bypass for management of CSSS especially with concomitant critical carotid artery atherosclerotic disease.

## 1. Introduction

Coronary-subclavian steal syndrome (CSSS) is defined as a reversal of flow in a previously constructed internal mammary artery (IMA) coronary conduit, producing myocardial ischemia. This is typically caused by proximal subclavian artery stenosis in patients with an ipsilateral IMA coronary conduit [1]. This condition may have broad spectrum of symptoms related to alteration in flow in coronary circuit as well as changes in cerebrovascular hemodynamics. Posterior cerebral circulation [2] can be adversely affected as well as the ipsilateral brachial artery. 

CSSS may be treated endoscopically with stents if the lesion can be ameliorated, otherwise surgery is required which may include aortosubclavian bypass [3], carotid subclavian bypass [4], or axilloaxillary [5] bypass techniques. We are presenting a case which had an unusual presentation of CSSS, denied any chest pains to the presenting physician, and had mainly syncopal symptoms. He had a complicated hospital course limiting his medical management but finally had a successful subclavian-subclavian bypass.

## 2. Case Report

An 80-year-old Caucasian male with history of coronary artery bypass graft (CABG) was brought to emergency room (ER) after being found unconscious at home. Detailed examination showed that left radial pulse was markedly diminished compared to right side and blood pressure on left arm was 60/44. There was numbness and weakness of left hand with hand exercise but did not result in any overt mental status changes or cardiovascular manifestations. No bruit was appreciated over the subclavian artery; however, carotid bruit was appreciated bilaterally. Electrocardiogram showed sinus rhythm with RSR pattern in V1, Q waves in V2, V3, with T wave inversions in AVL, which were new compared to his old EKG. Troponin I was 22.75 ng/mL, and diagnosis of NSTEMI was made.

Eptifibatide was started per cardiac and renal protocol. The patient had chronic kidney disease with acute worsening due to dehydration. Echocardiogram showed anterior wall, distal septal, and apical hypokinesia with ejection fraction around 50%, grade II diastolic dysfunction, moderate mitral and tricuspid regurgitation, and severe pulmonary hypertension. Difference in blood pressure in two arms was indicative of underlying left subclavian artery stenosis which was probably the cause of his syncope. Cardiac catheterization was planned, but patient was at considerable risk for contrast induced nephropathy given his renal insufficiency.

**Figure 1:**
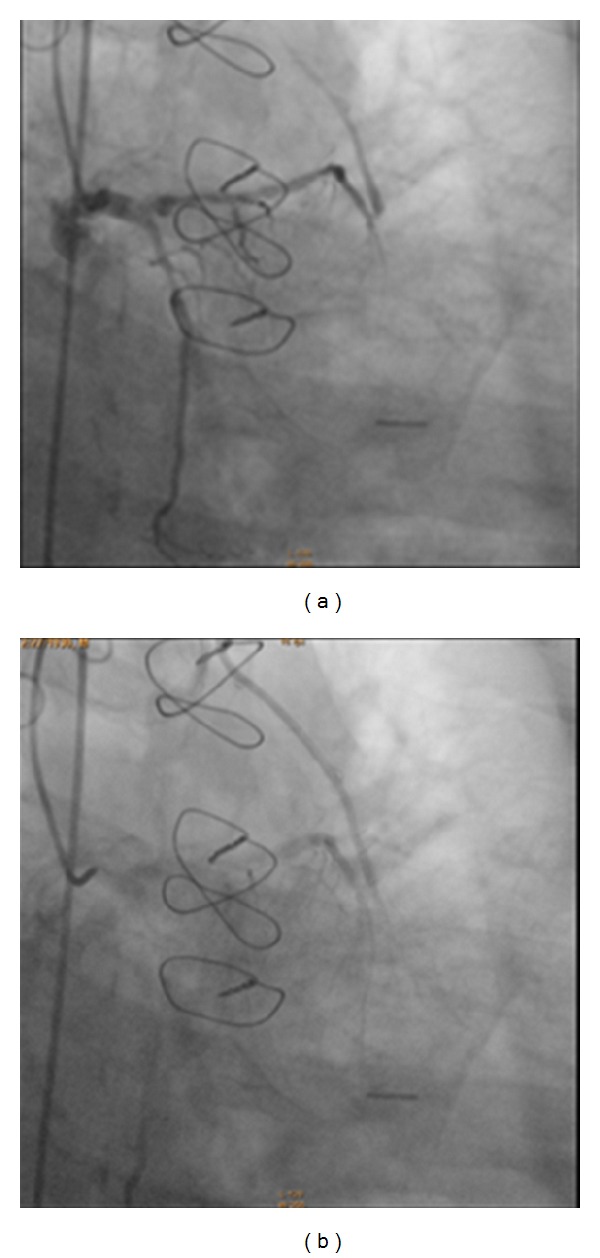
Reversal of flow of dye in left internal mammary artery graft to left anterior descending artery.

Patient had left heart catheterization after stabilization of renal insufficiency. This showed that left main coronary artery bifurcated into the left anterior descending and left circumflex artery. The left main coronary artery was normal. The left anterior descending coronary artery (LAD) showed an ostial 90–95% stenosis. The proximal LAD was diffusely diseased, and mid LAD was 100% occluded. There was evidence of flow into the left internal mammary artery (LIMA) anastomosis to LAD, distal to mid LAD occlusion. There was significant back filling of the LIMA graft with minimal competitive flow (Figures 1(a) and 1(b)). The first obtuse marginal artery (OM1) was 100% occluded. The right coronary artery was proximally occluded 100%. The saphenous vein graft (SVG) to the right coronary artery was patent. The SVG to the OM1 was widely patent and provided grade III collaterals to the LAD system. The LIMA to the LAD was not cannulated; however, the graft itself appeared to be healthy based on back filling with left coronary injection. Pressure in the LIMA appeared low.

**Figure 2:**
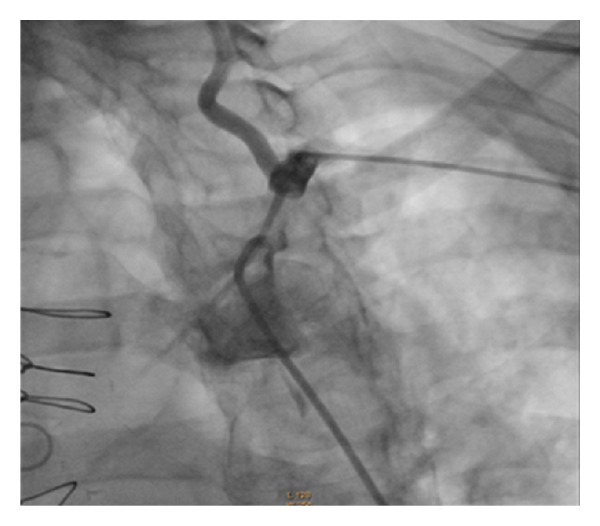
Absence of flow in proximal left subclavian artery with dual dye injection from catheters in aorta and left radial artery.

Selective injection into the left subclavian artery demonstrated high-grade stenosis of 99% or greater in the ostial left subclavian artery (Figure 2). Diagnosis of coronary subclavian steal syndrome (CSSS) was made.

Angioplasty confirmed 2.5 cm long calcified occlusion of the left subclavian artery. It extended from the aortic arch to the near vertebral artery. A 6 French R4 guide was advanced and engaged into the ostium of the left subclavian artery. An attempt was made to cross this stenosis with a Terumo Glide followed by a stiff Terumo Glide. 4000 units of heparin were given. The obstruction was approached distally using a 5 French R4 guide and a Terumo Glide followed by a stiff Terumo Glide. Attempts were unsuccessful to cross this lesion. The procedure was aborted due to severe calcification and length of stenosis.

Cardiothoracic surgery was consulted for possibility of bypass surgery to ameliorate the stenosed section of subclavian artery. Carotid duplex was obtained which showed evidence of bilateral carotid artery stenosis of 70–90% on the right and 50–69% on the left. Given the bilateral carotid artery disease, carotid subclavian bypass was deferred.

**Figure 3:**
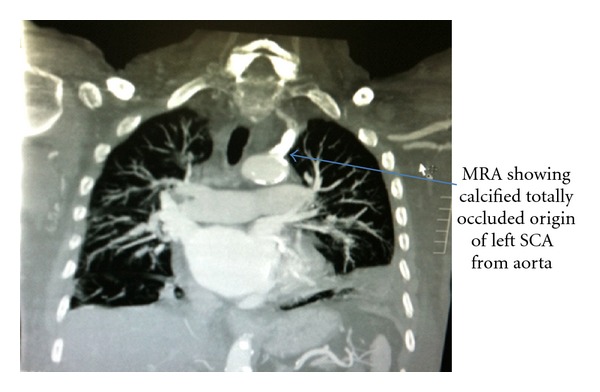
Magnetic resonance angiography showing completely occluded proximal left subclavian artery.

Later on, the patient had magnetic resonance angiography (Figure 3) which delineated his coronary and subclavian anatomy appropriately, and he finally underwent a bypass to his proximal left subclavian artery stenosis from the right subclavian artery (subclavian-subclavian bypass).

**Figure 4:**
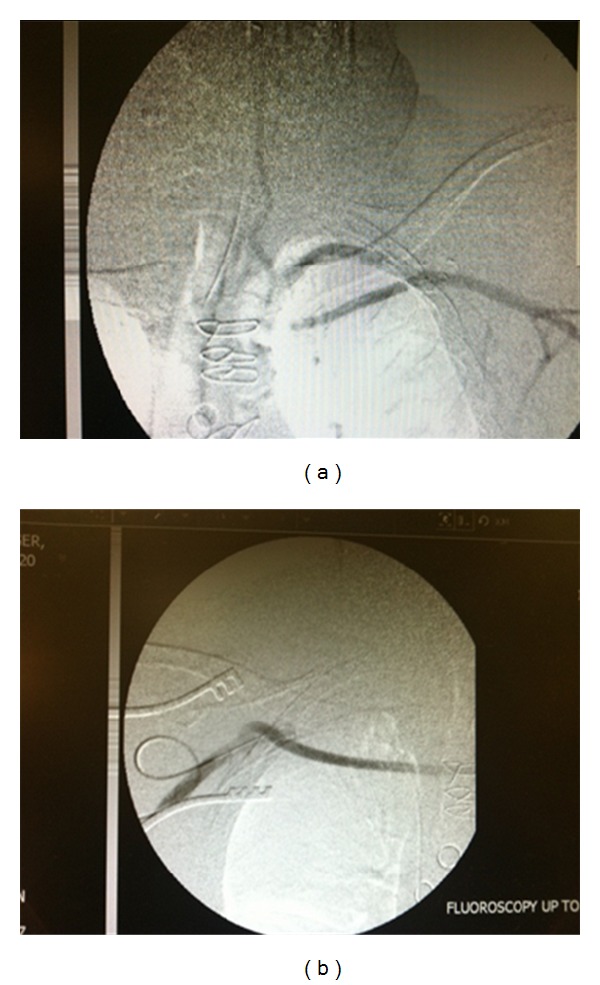
Restoration of flow in the left subclavian artery across a propaten graft from the right subclavian artery.

Surgery was performed using propaten graft to bridge the patent portion of left SCA with right SCA. Completion angiogram showed that the graft was widely patent, that the left subclavian artery did show flow from the right side, and that there was an antegrade flow reestablished into the vertebral artery (Figures 4(a) and 4(b)).

This was a daring attempt on the part of cardiothoracic surgeon which resulted in complete amelioration of his symptoms and normalized blood pressure in both arms thus decreasing potential of further cardiovascular or cerebrovascular adverse events. His postoperative course was uneventful. 

## 3. Discussion

Coronary subclavian steal syndrome is a rare complication of CABG. Stenosis of left subclavian artery prior to bifurcation of vertebral artery is central in pathophysiology of this syndrome. Any CABG candidate having difference in systolic blood pressure between the two arms of 20 mmHg should have presumptive diagnosis of subclavian artery stenosis, and this lesion be revascularized prior to CABG.

 The incidence of significant brachiocephalic disease in patients who undergoes elective CABG is 0.5% to 2.0% [6], a more recent study reports that the incidence of concomitant disease is 0.1% to 0.2% [3]. After CABG, this stenosis may become progressive and cause significant impairment of forward flow across lesion resulting in reversal of flow in LIMA and therefore becoming symptomatic. Other than atherosclerotic heart disease, possible causes of CSSS include Takayasu arteritis, radiation arteritis, and giant cell arteritis [1].

Our case report highlights the poor quality of life and real risk of recurrent complications in patients suffering from CSSS. Myocardial ischemia and risk of defective circulation in posterior cerebral circulation are hallmark of this syndrome. Therefore, endeavoring to ameliorate this condition is desirable either endovascular or by surgical bypass techniques.

In our patient, both these routes were tried sequentially. Literature is full of kudos for endovascular intervention to open SCA occlusion with relatively little complications. This approach was tried in our patient, but the lesion could not be threaded secondary to length of lesion and high degree of calcification indicating the chronicity of occlusion.

Surgery is the definitive route to fix CSSS. Carotid-subclavian bypass is the most widely used surgical intervention; other less common options are available depending on the patient's anatomy. However, our patient had significant carotid artery disease making this option less feasible. Finally it was decided to perform subclavian to subclavian bypass which involved putting a graft to connect left with right SCA bypassing the totally occluded portion of left SCA.

## Abbreviations

CAD: Coronary artery disease
CSSS: Coronary subclavian steal syndrome
LIMA: Left internal mammary artery
SVG: Saphenous venous graft
LAD: Left anterior descending artery
RCA: Right coronary artery
SCA: Subclavian artery
OM: Obtuse marginal artery
PTA: Percutaneous transluminal angioplasty
CABG: Coronary artery bypasses grafting
MRA: Magnetic resonance angiogram.
